# Short-term dietary restriction in old mice rejuvenates the aging-induced structural imbalance of gut microbiota

**DOI:** 10.1007/s10522-019-09830-5

**Published:** 2019-08-10

**Authors:** Ting Zeng, Hui Cui, Duozhuang Tang, George B. Garside, Yiting Wang, Jianying Wu, Zhendong Tao, Liu Zhang, Si Tao

**Affiliations:** 1 Jiangxi Key Laboratory of Clinical and Translational Cancer Research, Department of Oncology, The Second Affiliated Hospital of Nanchang University, Jiangxi, China; 2 Department of Oncology, The Second Affiliated Hospital of Nanchang University, Min-De Road. 1, 330006 Nanchang City, Jiangxi Province China; 3 Department of Hematology, The Second Affiliated Hospital of Nanchang University, Jiangxi, China; 4 Leibniz Institute on Aging - Fritz Lipmann Institute (FLI), Jena, Germany; 5 Department of Medical Laboratory Medicine, Jiangxi Province Hospital of Integrated Chinese & Western Medicine, Jiangxi, China; 6 Intensive Care Unit, Peking University People’s Hospital, Beijing, China

**Keywords:** Aging, Dietary restriction, Gut microbiota, Rejuvenate, Obesity, Inflammation

## Abstract

The world’s aging population is growing rapidly. Incidences of multiple pathologies, such as abdominal obesity, cardiovascular and cerebrovascular diseases, type 2 diabetes, and malignant neoplasms, increase sharply with age. Aged individuals possess a significantly shifted composition of gut microbiota, which is suggested to play important roles in aging associated pathologies. Whether the existing shifted structural composition of microbiota in aged populations can be reverted non-pharmacologically has not been studied so far. Here, we show an intestinal flora imbalance in old C57BL/6J mice with a remarkable dominant proportion of microbes promoting lipid metabolism and inflammation. Intriguingly, short-term (2 months) dietary restriction was enough to significantly revert the imbalance of intestinal flora in aged mice toward a more balanced structural composition as shown in young mice. Our study provides the first evidence that short-term dietary restriction in old mice can restore the already dysfunctional aged gut microbiota. Our study provides the first evidence that short-term dietary restriction in old mice can restore the already dysfunctional aged gut microbiota, which may help ameliorate aging-related disorders plaguing the vast elderly population.

## Introduction

The trend towards an aging population is increasing, with the 2017 United Nations World Population Prospects report revealing that people over 60 years old will make up more than 25% of a nation’s population in the near future in many countries. Biological aging involves molecular and physical changes that increase the probability of developing certain diseases such as osteoporosis, arthritis, type 2 diabetes, hypertension, heart disease, and cancer. While the underlying mechanisms remain incompletely understood, it is generally believed to involve a progressive decline in organ functionality and tissue homeostasis, which is suggested to be closely related to microbiota shifts (Claesson et al. 2012; Liu and Rando 2011; Zapata and Quagliarello 2015). The number of bacteria in the body is estimated to be as much as 10 trillion, with the vast majority of bacteria residing in the colon. Imbalance of the intestinal microbiota can lead to increased permeability of the intestinal wall and dysfunction of the intestinal mucosal barrier (Bischoff et al. 2014; Clark et al. 2015; Thevaranjan et al. 2017). Furthermore, the intestinal microbiota can regulate lipid uptake and storage by invading intestinal mucosal cells and altering their circadian rhythm, contributing to the development of metabolic diseases, such as obesity, diabetes, and nonalcoholic fatty liver (Amar et al. 2011; Boursier et al. 2016; Wang et al. 2017).

It has been shown that the composition of the gut microbiota shifts towards increasing proinflammatory commensals and decreasing beneficial commensals microbes with age in both humans and rodents (Biagi et al. 2016; Biragyn and Ferrucci 2018; Hopkins et al. 2001; Jeffery et al. 2016), which is accompanied by an impairment of the intestinal barrier (Clark et al. 2015). Furthermore, gut dysbiosis contributes to a chronic proinflammatory state and could serve as a potential link between cancer risk in aging (Biragyn and Ferrucci 2018). Interventions that shift the gut microbiota towards a younger state might therefore help ameliorate aging associated pathologies. Recent studies have found that high sugar and high fat diet-induced obesity leads to changes in the composition of the intestinal microflora (Carmody et al. 2015; He et al. 2018). Obesity in humans and mice has also been found to be positively correlated with the ratio of Firmicutes/Bacteroidetes (F/B) (He et al. 2018; Ley et al. 2005, 2006; Turnbaugh et al. 2006, 2009). Studies have shown that changes in the composition of the gut microbiota in the elderly are not only related to chronic diseases such as obesity and inflammation, but also have a significant relationship with diet (Claesson et al. 2012). Dietary intervention has a great impact on the intestinal microbiota (Cotillard et al. 2013), a wealth of evidence suggest that dietary restriction (DR) has wide-ranging benefits in increasing the body’s general health status and in providing a nonspecific resistance to chronic diseases and metabolic derangements (Colman et al. 2009; Fontana et al. 2004; Ribaric 2012; Roth et al. 2001; Walford et al. 2002). Previous studies have explored the role of life-long DR on gut microbiota. Life-long DR, in combination with low-fat diet, maintained a structurally balanced architecture of the gut microbiota and improved colonic health (Kok et al. 2018; Zhang et al. 2013). However, DR was already initiated from young age when the gut microbiota was still healthy in all the above-mentioned studies. Whether, the existing imbalanced structural composition of microbiota in aged populations can be reverted non-pharmacologically has yet to be studied. Exploring the possibilities of how the established gut microbiota in aged organisms might be reformed could benefit the health of the growing elderly population.

In this study, we performed 16S rRNA gene sequencing of bacterial DNA extracted from freshly collected faecal samples to examine the effects of DR on the microbiota of aging mice. In line with previous studies, we found that the composition of the intestinal flora in old mice (20–22 months old) mainly shifted towards a proinflammatory state and promotion of lipid metabolism. Intriguingly, short-term (2 months) DR (dietary restriction) performed in aged mice significantly reverted the intestinal flora imbalance towards a more balanced structural composition as shown in young mice. This structural imbalance shown in old mice was rejuvenated by reducing the dominance of Clostridia, Clostridiales, and Firmicutes which have all been found to contribute to obesity and inflammation. Our study provides the first evidence that short-term DR in old organisms can rejuvenate the imbalanced composition of gut microbiota. Therefore, we identified a non-pharmacological and efficient way to rejuvenate gut microbiota in aged mice, which may benefit health in the elderly population.

## Results

### Bacterial community shifts towards dominant lipid-promoting and pro-inflammatory bacteria in aging intestine

To study whether microbiota composition changes upon aging, we collected fecal samples from young (2 months old) and old (20–22 months old) female mice. Hypervariable regions were sequenced by 16S rRNA high-throughput sequencing platform Illumina Hiseq 2500. 1,127,979 sequence reads were generated from the 16S rRNA gene V4 amplicon, with an average reading of 32,228 (± 2024 SEM) per subject.

**Fig. 1:**
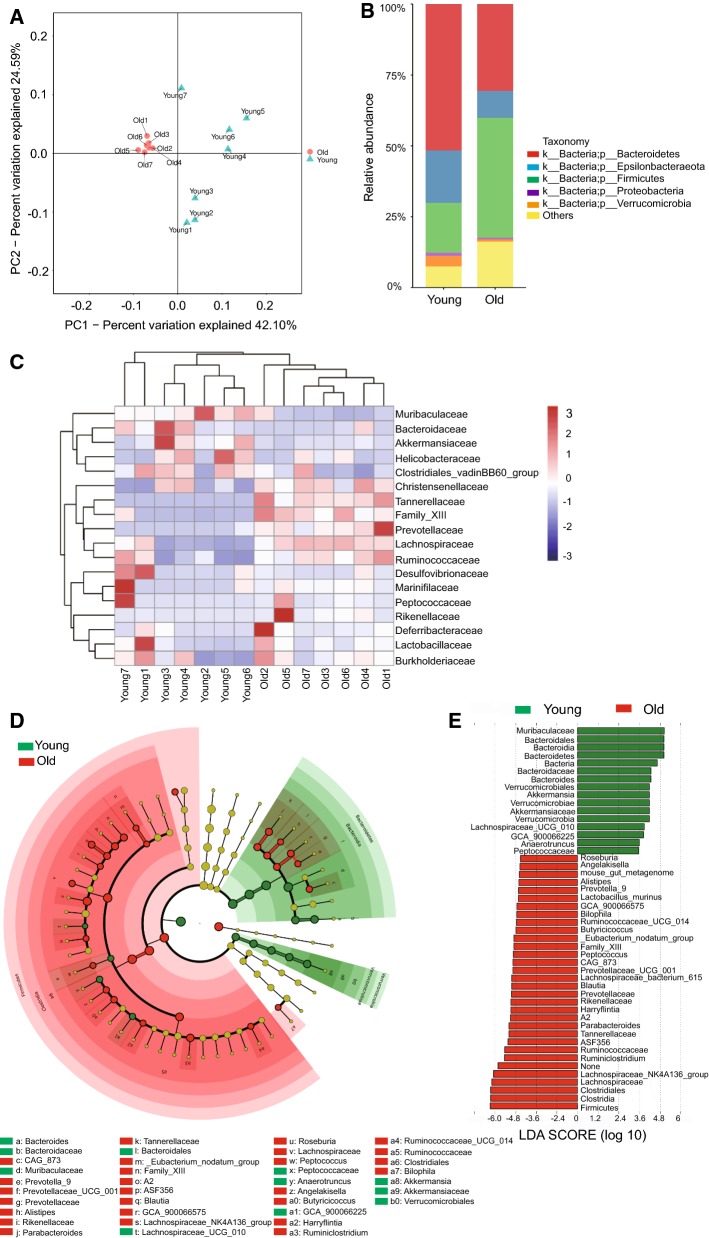
Alterations in the fecal microbial community structure of aging mice. Fecal samples of 2 months old (young) and 20–22 months old (old) mice were collected for analysis (n = 7 samples per group). **a** β-diversity analysis. The results of unweighted UniFrac PCoA were shown. **b** Relative abundance of bacteria at phylum level. The ratio of the average OTU for each group was shown. **c** Heatmap based on the relative abundance at family level. **d** Taxonomic cladogram from LEfSe showing differences in fecal taxa. Dot size is proportional to the abundance of the taxon. Letters correspond to the following taxa: a: Bacteroides, b: Bacteroidaceae, c: CAG_873, d: Muribaculaceae, e: Prevotella_9, f: Prevotellaceae_UCG_001, g: Prevotellaceae, h: Alistipes, i: Rikenellaceae, j: Parabacteroides, k: Tannerellaceae, l: Bacteroidales, m: _Eubacterium_nodatum_group, n: Family_XIII, o: A2, p: ASF356, q:Blautia, r: GCA_900066575, s: Lachnospiraceae_NK4A136_group, t: Lachnospiraceae_UCG_010, u: Roseburia, v: Lachnospiraceae, w: Peptococcus, x: Peptococcaceae, y: Anaerotruncus, z: Angelakisella, a0: Butyricicoccus, a1: GCA_900066225, a2: Harryflintia, a3: Ruminiclostridium, a4: Ruminococcaceae_UCG_014, a5: Ruminococcaceae, a6: Clostridiales, a7: Bilophila, a8: Akkermansia, a9: Akkermansiaceae, b0: Verrucomicrobiales. **e** LDA scores computed for differentially-abundant taxa in the fecal microbiomes of young and old mice. Length indicates effect size associated with a taxon. p = 0.05 for the Kruskal–Wallis test; LDA score > 2

β-diversity analysis via the principal coordinate analysis (PCoA) analysis based on the Unweighted Unifrac distance indicated a clear separation between young intestinal flora and the old intestinal flora (Fig. 1a), indicating that the bacterial community was significantly changed upon aging. Analysis on the phyla level showed that Bacteroidetes was the most dominant phyla in young mice while it was significantly reduced in old mice (Fig. 1b). Epsilonbacteraeota and Firmicutes were the second most dominant phyla with an almost equal ratio in young mice, while Firmicutes increased to be the most abundant phyla in old mice (Fig. 1b). Bacterial taxa ranking at the top of the list in young mice all fell from the top positions in old mice; further analysis showed that families of Christensenellaceae, Family_XIII, Prevotellaceae, Lachnospiraceae, and Ruminococcaceae were all enriched in the gut microbiota of aged mice, while Bacteroidaceae and Muribaculaceae were enriched in the microbiota of young mice (Fig. 1c). To identify the most differently abundant taxa in aged mice, we used the linear discriminant analysis (LDA) effect size (LEfSe) method to assess the effect size of each taxon (Fig. 1e, f). In young mice, the intestinal flora was mainly enriched with Muribaculaceae, Bacteroidales, Bacteroidetes, Bacteroidaceae and Bacteroides, with all of these falling from the top-ranking positions in old mice. Old mice were comparatively enriched with Clostridia, Clostridiales and Firmicutes. The analysis further proved a significantly altered composition of gut microbiota in old mice compared to the young ones.

**Fig. 2:**
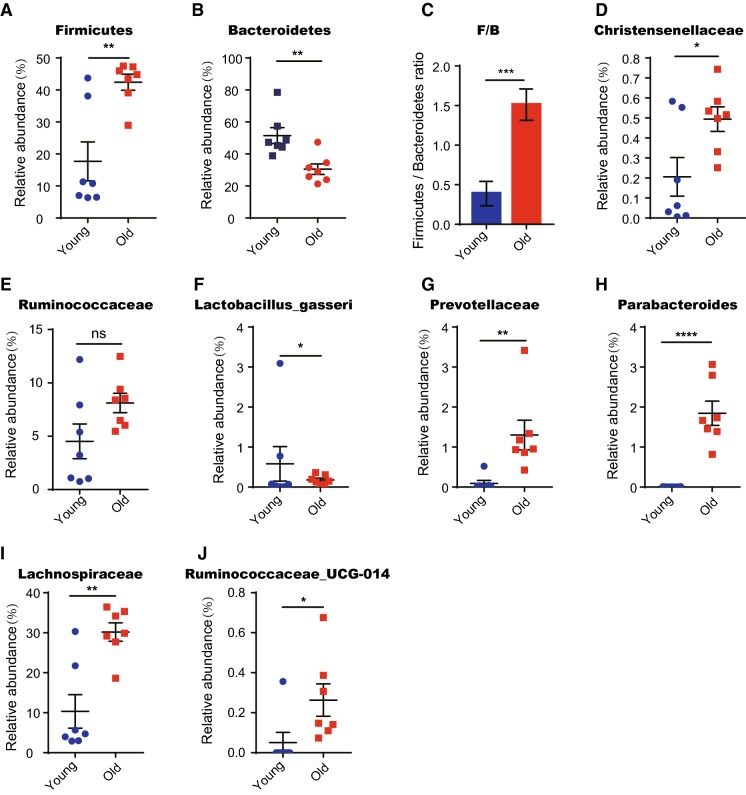
Lipid-promoting and pro-inflammatory bacteria are enriched in aging mice. Fecal samples of 2 months old (young) and 20–22 months old (old) mice were collected for analysis (n = 7 samples per group). **a**, **b**, **d**–**j** Relative abundance based on OTUs of intestinal bacteria taxa which are significantly changed in old mice. Note that these taxa were all lipid-promoting or pro-inflammatory bacteria. **c** Ratio of Firmicutes/Bacteroidetes based on relative abundance of OTUs. Note a significant increase in the old mice compared to the young ones. Results were displayed as mean ± SEM. *p < 0.05; **p < 0.01; ***p < 0.001; ****p < 0.0001 by unpaired two-tailed Student’s t test

The Firmicutes/Bacteroidetes (F/B) ratio has been associated with obesity in humans and mice (He et al. 2018; Ley et al. 2005, 2006; Turnbaugh et al. 2006, 2009). Here we show that the proportion of Firmicutes was 18 ± 6.0% in young mice versus 42 ± 2.5% in old mice (Fig. 2a). Conversely, the proportion of Bacteroidetes decreased significantly from 52 ± 4.9% in young mice to 31 ± 3.3% in old mice (Fig. 2b). Therefore, the Firmicutes/Bacteroidetes ratio (F/B) in old mice was significantly higher than young mice (1.5 ± 0.2 in old mice vs. 0.4 ± 0.1 in young mice) (Fig. 2c). Interestingly, our study determined a significant increase of Ruminococcaceae and Christensenellaceae, and a decrease of *Lactobacillus gasseri* in old mice (Fig. 2d–f). These alterations in the structural microbiota composition in aged mice were reported to be positively related to fat accumulation (Bauer et al. 2018; Qin et al. 2018). Prevotellaceae, Parabacteroides, Oscillibacter, Lachnospiraceae, Ruminococcaceae-UCG-014 and Erysipelatoclostridium have all been reported to be increased in multiple inflammatory models, and whose related bacterial taxa were also enriched in aged mice (Fig. 2g–j) (Elinav et al. 2011; Peng et al. 2019; Qi et al. 2019). Taken together, the results indicate that aging leads to alterations in intestinal flora composition that have previously been positively correlated to lipid accumulation and inflammation.

### Short-term DR in old mice rejuvenates aging induced structural imbalance of gut microbiota

In line with previous studies, the above data indicate a clear aging phenotype involving compositional change of the gut microbiota. To investigate whether DR can rejuvenate the aged gut microbiota, we treated 20–22 months old female mice with 30% DR for 2 months and examined the composition of intestinal flora compared to identically aged female mice fed ad libitum (AL) as well as AL fed 2 months old mice.

**Fig. 3:**
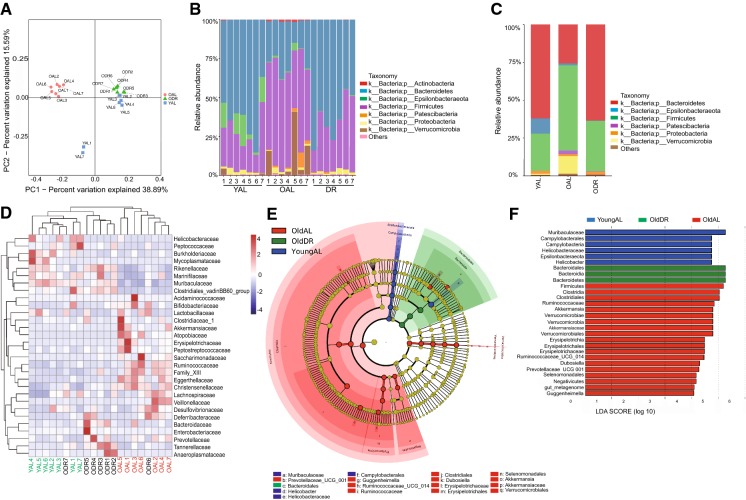
Short-term DR in old mice rejuvenates aging induced structural rearrangement of gut microbiota. 20–22 months old mice were treated with DR or AL diet for 2 months. Fecal samples of the following groups were collected for analysis: YAL (2 months old mice fed with ad libitum), OAL (22–24 months old mice fed with ad libitum), and ODR (22–24 months old mice pre-treated with DR for 2 months before sample collection) (n = 7 samples per group). **a** β-diversity analysis. The results of unweighted UniFrac PCoA of indicated groups were shown. **b** Relative abundance of bacteria at phylum level of individual sample based on OTUs. **c** The ratio of relative abundance at phylum level based on the average OTUs in each group. **d** Heatmap showing clustering of each sample at family level based on the relative abundance of OTUs. Note that hierarchical clustering shown that samples of ODR and YAL tend to cluster together. **e** Taxonomic cladogram from LEfSe showing differences in fecal taxa. Dot size is proportional to the abundance of the taxon. Letters correspond to the following taxa: a: Muribaculaceae, b: Prevotellaceae_UCG_001, c: Bacteroidales, d: Helicobacter, e: Helicobacteraceae, f: Campylobacterales, g: Guggenheimella, h: Ruminococcaceae_UCG_014, i: Ruminococcaceae, j: Clostridiales, k: Dubosiella, l: Erysipelotrichaceae, m: Erysipelotrichales, n: Selenomonadales, o: Akkermansia, p: Akkermansiaceae, q: Verrucomicrobiales. **f** LDA scores computed for differentially-abundant taxa in the fecal microbiomes of young (blue) old DR (green) and old AL (red). Length indicates effect size associated with a taxon. p = 0.05 for the Kruskal–Wallis test; LDA score > 2

We first performed a diversity analysis among the three groups: young AL (2 months old mice fed with ad libitum), old AL (22–24 months old mice fed with ad libitum), and old DR (22–24 months old mice fed with DR for 2 months before analysis). The β-diversity analysis, PCoA analysis based on Unweighted Unifrac distance showed that old AL samples were clearly separated from the young AL samples, while the old DR samples were not. This finding demonstrates that the significantly altered composition and structure of an aged biological community can be reverted via short-term DR (Fig. 3a).

Further analysis on the phyla level showed that the structure of the biological community of short-term DR mice converged with that of young AL mice, which was clearly different compared to old AL mice. In detail, Firmicutes was the most abundant phyla in the old AL mice, which was significantly decreased in young AL and old DR mice. The most abundant phyla in young AL and old DR mice was Bacteroidetes, which was clearly reduced in the old AL mice (Fig. 3b, c). At the family level, the composition and structure of the intestinal flora was similar in young AL and old DR mice, while it was distinct from the old AL mice (Fig. 3d). The LEfSe method was used to further assess the effect size of each taxon (Fig. 3e, f). The analysis showed that old DR mice had more Bacteroidales, Bacteroidia and Bacteroidia, with less Firmicutes, Clostridia and Ruminococcaceae, which is further evidence of microbiota rescue in aged individuals achieved by short-term DR (Fig. 3f).

**Fig. 4:**
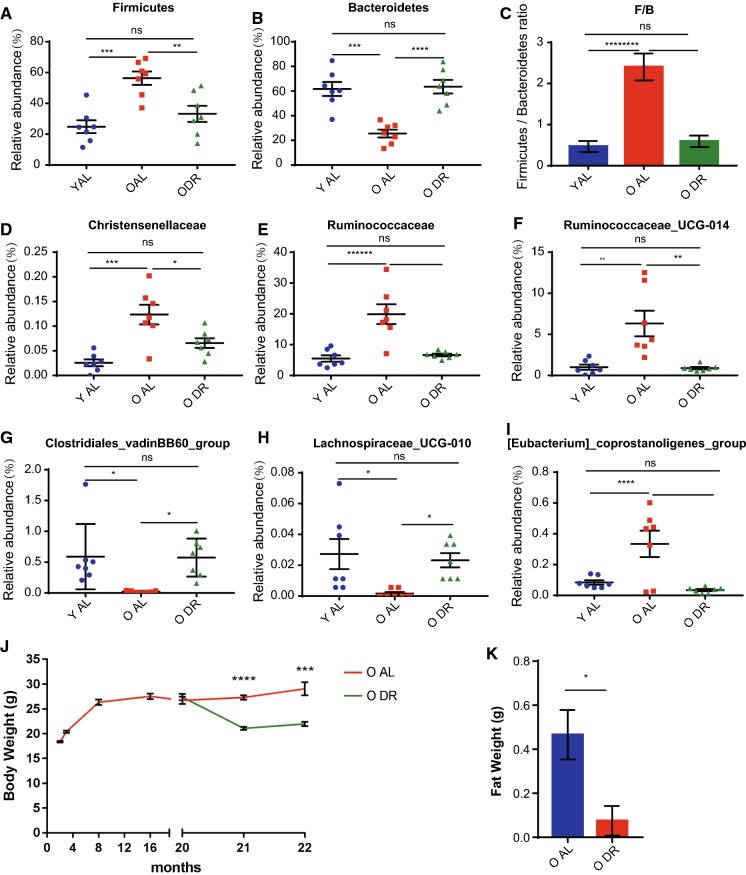
Short-term DR reverted compositional alterations of bacterial taxa associated with obesity and inflammation in aging mice. 20–22 months old mice were treated with DR or AL diet for 2 months. Fecal samples of the following groups were collected for analysis: YAL (2 months old mice fed with ad libitum), OAL (22–24 months old mice fed with ad libitum), and ODR (22–24 months old mice pre-treated with DR for 2 months before sample collection) (n = 7 samples per group). **a**, **b**, **d**–**i** Relative abundance based on OTUs of intestinal bacteria taxa. **c** Ratio of Firmicutes/Bacteroidetes based on relative abundance of OTUs. Note that DR significantly rejuvenated all alterations of indicated taxa in aging mice. **j**, **k** Body and belly fat weight of indicated groups. Note a significant reduction upon DR. Results were displayed as mean ± SEM. *p < 0.05; **p < 0.01; ***p < 0.001; ****p < 0.0001 by unpaired two-tailed Student’s t test

We further determined the effect of DR on bacterial taxa that were functionally relevant to obesity and inflammation. Firmicutes was the most abundant phyla in the old AL mice (56 ± 4.4%), while it was significantly less in young AL (25 ± 4.2%) and old DR mice (33 ± 5.3%) (Fig. 4a). The most abundant phyla in young AL and old DR mice was Bacteroidetes (62 ± 5.8% in young AL and 63 ± 5.5% in old DR), while it was clearly reduced in the old AL mice (26 ± 3.1%) (Fig. 4b). The F/B ratio in old AL mice was 2.4 ± 0.3, which was significantly reduced in the old DR mice to 0.6 ± 0.1, close to the level in the young mice at 0.5 ± 0.1 (Fig. 4c). Of note, short-term DR reverted almost all compositional changes of bacterial taxa associated with obesity and inflammation and brought their level close to that of young mice, including Bacteroidetes, Firmicutes, Christensenellaceae, Ruminococcaceae, and Ruminococcaceae_UCG_014 (Fig. 4a–f). Intriguingly, short-term DR additionally resulted in changes in more bacterial taxa that were reported to contribute to fat accumulation and inflammation, such as Clostridiales_vadinBB60_group, Lachnospiraceae_UCG-010, [Eubacterium]coprostanoligenes_group (Fig. 4g–i). These findings indicate that short-term DR was enough to induce changes in the composition of the commensal community towards a younger, healthier state which in turn may associate with less fat accumulation and decreased inflammation. We also note that short-term DR expectedly reduces the body weight and abdominal fat in old mice compared to old ad libitum fed mice (Fig. 4j, k).

## Discussion

While lifelong DR has been proven to be a robust regimen to retard aging in many animal models, it is unpractical in humans (Colman et al. 2009; Goto 2006; Goto et al. 2007; Mattison et al. 2012; Weindruch 1996). Therefore, a number of studies considered the effects of DR initiated in later life and showed that late-onset DR could rejuvenate biological parameters that decline with age in rodents, such as improved protein and lipid metabolism and chromatin functions. These improvements in turn protect the functionalities of different organs, including the brain, skeletal muscle, and immune system (Goto 2006; Goto et al. 2007; Radak et al. 2002; Singh et al. 2012, 2015; Weindruch et al. 1982). However, the underlying mechanisms remains to be further elucidated.

The gut microbiota has been shown to play essential roles in multiple biological processes as well as pathologies. The current study described a significant structural rearrangement of gut microbiota in old mice (20–22 months old). The skewed intestinal flora has potential in promoting lipid accumulation and inflammation; therefore, it could be highly relevant to associated pathologies in aging. Whether and how the already aged microbiota could be rejuvenated has never been studied so far. Here, we provide the first experimental evidence that short-term (2 months) DR in old mice was enough to revert the already skewed gut microbiota. The equivalent change in humans would roughly be a 66–72 year old’s intestinal flora reverting back to a similar state to when they were around 23 years’ old (Dutta and Sengupta 2016). Our study showed that old mice receiving short-term DR exhibit a younger-shaped intestinal flora along with reduced body weight and abdominal fat. Assessing any potential contribution of a more balanced microbiome in old mice to weight loss will be an important topic of further research, especially given recent evidence demonstrating the rescue of DR-induced weight loss by fecal transplantation from AL mice (Wang et al. 2018).

Recent studies have found that obesity in humans and mice is primarily associated with changes in the relative abundance of Bacteroidetes and Firmicutes, and is positively correlated with the ratio of Firmicutes/Bacteroidetes (F/B) (He et al. 2018; Ley et al. 2005, 2006; Turnbaugh et al. 2006, 2009).Therefore, the elevated F/B ratio we observed in aged mice might serve as an underlying mechanism why fat accumulates with age in general. In line with previous studies, our study uncovered aging-associated changes of the gut microbiota, including increases of Firmicutes, Ruminococcaceae and Christensenellaceae, and decreases of Bacteroidetes and *Lactobacillus gasseri*. Interestingly, it was previously shown that Ruminococcaceae and Christensenellaceae are enriched in obese mice (Qin et al. 2018), and that *Lactobacillus gasseri* levels were reduced in high-fat diet mice (Bauer et al. 2018). Our study shows that short-term DR can reverse the imbalanced microbial community structure of aged mice and restore the proinflammatory and lipid metabolism promoting intestinal flora to a young level, which may reduce the incidence of age-related diseases.

Previous studies have shown that androgens influence gut microbiota which differs between males and females (Haro et al. 2016; Markle et al. 2013; Yurkovetskiy et al. 2013). While this study was performed on female mice for the benefit of co-housing before the dietary intervention, we would expect to observe the same rejuvenation effect of DR in old male mice. A recent assessment of calorie restriction (CR) noted that while its effect was invariably positive, the effect size could vary between mouse substrain, sex, and the extent of CR (Mitchell et al. 2016).

In this study we conclude that the existing proinflammatory and lipid-promoting microbiota in aged mice can be reverted non-pharmacologically to a young state using DR. This microbiota in old DR mice possesses a more balanced structural composition similar to that of young mice; the permanence and generalizability of this finding remains to be studied further.

## Materials and methods

### Animals and dietary intervention

C57BL/6 J female mice were obtained from Hunan SJA Laboratory Animal Co., Ltd. (Hunan, China) and maintained in the animal facilities of Nanchang Royo Biotech under pathogen-free conditions on a 12-h light/12-h dark cycle. 2 month old mice were used as a young cohort, and 20–22 month old mice used as an old cohort. Dietary interventions were performed according to a protocol from our previous publication (Tang et al. 2016). Briefly, bodyweight and age matched mice were randomly divided into either the AL-fed or DR-fed group. One week before the dietary intervention, mice were housed individually and daily food consumption was measured for every mouse to determine their AL-feeding rate. The average amount of food was determined after the 1-week measurement for every mouse. When initiating the feeding protocol, the AL mice were fed with unlimited access of food, while DR mice were fed with 70% the average amount of food according to the previous calculation. The calculated 70% food pellet was added to each cage daily at the same time, and was constant over the whole DR period. All mouse experiments were approved by the Animal Experimental Ethical Inspection of Nanchang Royo Biotech Co. Ltd (RYEI20170430-1).

### Sample collection

Fecal pellets were directly collected from each mouse in 1.5 ml microtubes by positioning the microtube in the proximity of the anus of the mouse and collecting the pellets that were excreted. All samples stored at − 80 °C until DNA isolation.

### Microbial DNA extraction, PCR amplification and Illumina Hiseq sequencing

DNA was extracted using DNA extraction kit (Minkagene Stool DNA kit) for the corresponding sample. The concentration and purity were measured using the NanoDrop One (Thermo Fisher Scientific, MA, USA). 16S rRNA genes of distinct region (V4) were amplified using specific primers (515F and 806R) with 12 bp barcodes. Primers were synthesized by Invitrogen (Carlsbad, CA, USA). PCR reactions, containing 25 μl 2 × Premix Taq (Takara Biotechnology, Dalian Co. Ltd., China), the PCR instrument was BioRad S1000 (Bio-Rad Laboratory, CA). The length and concentration of the PCR product were detected by 1% agarose gel electrophoresis. PCR products were mixed in equimolar ratios according to the GeneTools Analysis Software (Version4.03.05.0, SynGene). Then, the PCR mixture was purified with EZNA Gel Extraction Kit (Omega, USA). Then, sequencing libraries were generated using NEBNext® Ultra™ DNA Library Prep Kit for Illumina® (New England Biolabs, USA) following the manufacturer’s recommendations and index codes were added. The library quality was assessed on the Qubit 2.0 Fluorometer (Thermo Scientific) and Agilent Bioanalyzer 2100 system. Lastly, the library was sequenced on an Illumina Hiseq 2500 platform and 250 bp paired-end reads were generated.

### Bioinformatics and sequencing data analysis

Quality filtering on the paired-end raw reads were performed under specific filtering conditions to obtain the high-quality clean reads according to the Trimmomatic (V0.33, http://www.usadellab.org/cms/?page=trimmomatic) quality controlled process. At the same time, sequences were assigned to each sample based on their unique barcode and primer, after which the barcodes and primers were removed to get the paired-end clean reads. Paired-end clean reads were merged using FLASH (V1.2.11, https://ccb.jhu.edu/software/FLASH/) according to the relationship of the overlap between the paired-end reads, when at least 10 of the reads overlap the read generated from the opposite end of the same DNA fragment, the maximum allowable error ratio of the overlap region of 0.2, and the spliced sequences were called raw tags. Assigned Sequences to each sample based on their unique barcode and primer using Mothur software (V1.35.1, http://www.mothur.org), after which the barcodes and primers were removed to get the effective Clean Tags. Sequences analysis were performed by usearch software (V10, http://www.drive5.com/usearch/). Sequences with ≥ 97% similarity were assigned to the same OTU (Operational Taxonomic Units). An OTU is thought to possibly represent a species. The most frequently occurring sequence was extracted as representative sequence for each OTU and was screened for further annotation. For each representative sequence, the silva (for 16S, https://www.arb-silva.de/), database was used to annotate taxonomic information (set the confidence threshold to default to ≥ 0.5). The total number of otu sequences (No. of final seqs) and otu type (No. of OTUs) in otu_table were counted respectively. Based on the otu_table_subsampled, the annotation ratio on each classification level was calculated to obtain the sequence composition of each sample at each classification level. Based on the relative abundance of species at each classification level in otu_table, R software was used to draw the histogram, heat map and ternary phase diagram. Beta diversity analysis was used to evaluate differences of samples in species complexity. Beta diversity was determined using QIIME (Quantitative Insights Into Microbial Ecology) software. LDA Effect Size (LEfSe) analysis was used to find the biomarker of each group.

### Statistics

All statistical analyses were performed using GraphPad Prism 7.0 software. The unpaired two-tailed Student’s *t* test and One-way ANOVA were used to calculate p values. Data are expressed as mean ± SEM.
