# Correction to: Short-term dietary restriction in old mice rejuvenates the aging-induced structural imbalance of gut microbiota

**DOI:** 10.1007/s10522-019-09831-4

**Published:** 2019-09-09

**Authors:** Ting Zeng, Hui Cui, Duozhuang Tang, George B. Garside, Yiting Wang, Jianying Wu, Zhendong Tao, Liu Zhang, Si Tao

**Affiliations:** 1 Jiangxi Key Laboratory of Clinical and Translational Cancer Research, Department of Oncology, The Second Affiliated Hospital of Nanchang University, Jiangxi, China; 2 Department of Oncology, The Second Affiliated Hospital of Nanchang University, Min-De Road. 1, 330006 Nanchang City, Jiangxi Province China; 3 Department of Hematology, The Second Affiliated Hospital of Nanchang University, Jiangxi, China; 4 Leibniz Institute on Aging - Fritz Lipmann Institute (FLI), Jena, Germany; 5 Department of Medical Laboratory Medicine, Jiangxi Province Hospital of Integrated Chinese & Western Medicine, Jiangxi, China; 6 Intensive Care Unit, Peking University People’s Hospital, Beijing, China

## Correction to: Biogerontology 10.1007/s10522-019-09830-5

In the original publication of the article, the alphabets in figures were published in upper case and mismatched with the figure legends.

The corrected Figs. 1, 2, 3, 4 are given in this Correction.

**Fig. 1:**
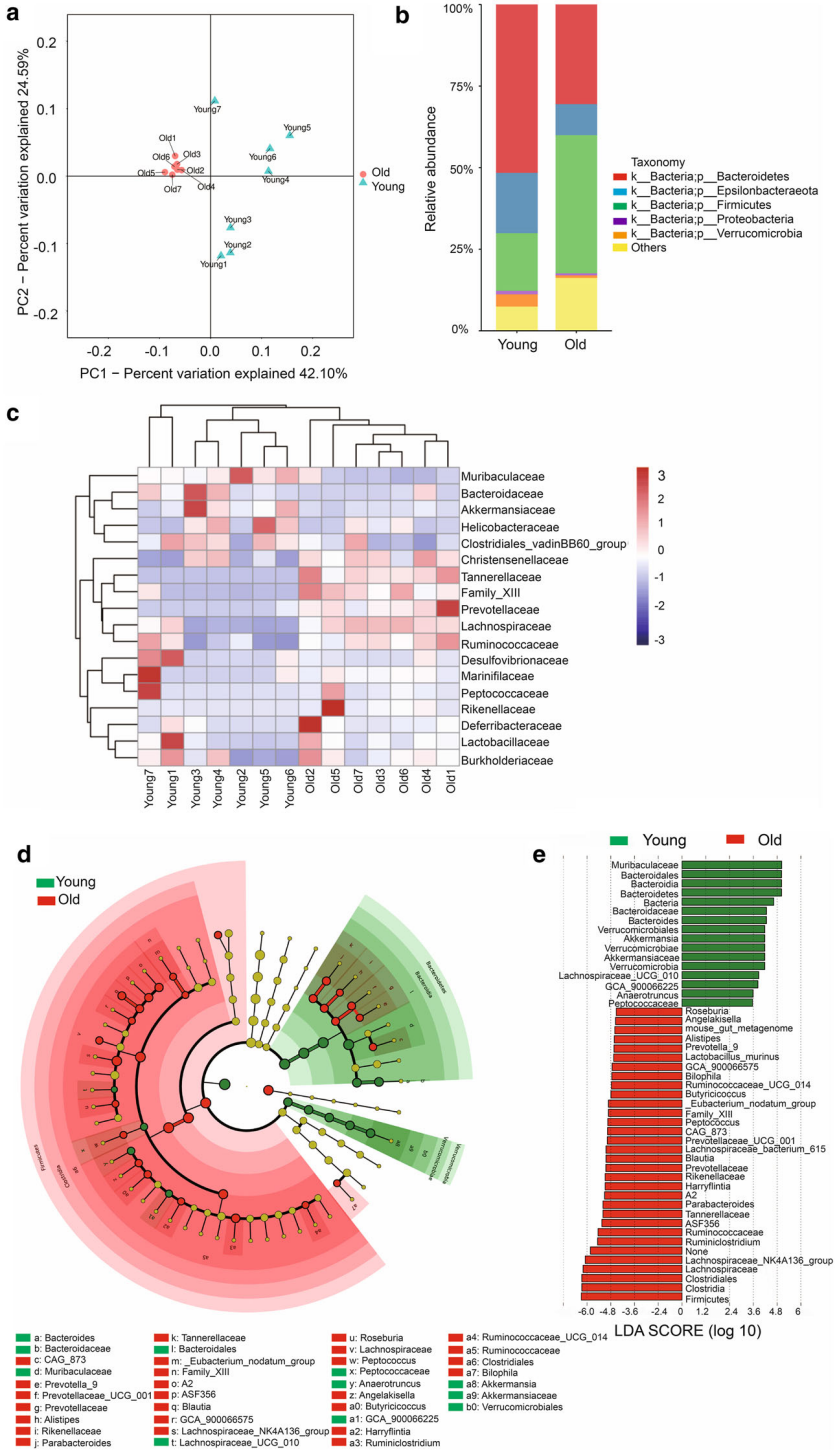
Alterations in the fecal microbial community structure of aging mice. Fecal samples of 2 months old (young) and 20–22 months old (old) mice were collected for analysis (n = 7 samples per group). **a** β-diversity analysis. The results of unweighted UniFrac PCoA were shown. **b** Relative abundance of bacteria at phylum level. The ratio of the average OTU for each group was shown. **c** Heatmap based on the relative abundance at family level. **d** Taxonomic cladogram from LEfSe showing differences in fecal taxa. Dot size is proportional to the abundance of the taxon. Letters correspond to the following taxa: a: Bacteroides, b: Bacteroidaceae, c: CAG_873, d: Muribaculaceae, e: Prevotella_9, f: Prevotellaceae_UCG_001, g: Prevotellaceae, h: Alistipes, i: Rikenellaceae, j: Parabacteroides, k: Tannerellaceae, l: Bacteroidales, m: _Eubacterium_nodatum_group, n: Family_XIII, o: A2, p: ASF356, q:Blautia, r: GCA_900066575, s: Lachnospiraceae_NK4A136_group, t: Lachnospiraceae_UCG_010, u: Roseburia, v: Lachnospiraceae, w: Peptococcus, x: Peptococcaceae, y: Anaerotruncus, z: Angelakisella, a0: Butyricicoccus, a1: GCA_900066225, a2: Harryflintia, a3: Ruminiclostridium, a4: Ruminococcaceae_UCG_014, a5: Ruminococcaceae, a6: Clostridiales, a7: Bilophila, a8: Akkermansia, a9: Akkermansiaceae, b0: Verrucomicrobiales. **e** LDA scores computed for differentially-abundant taxa in the fecal microbiomes of young and old mice. Length indicates effect size associated with a taxon. p = 0.05 for the Kruskal–Wallis test; LDA score > 2

**Fig. 2:**
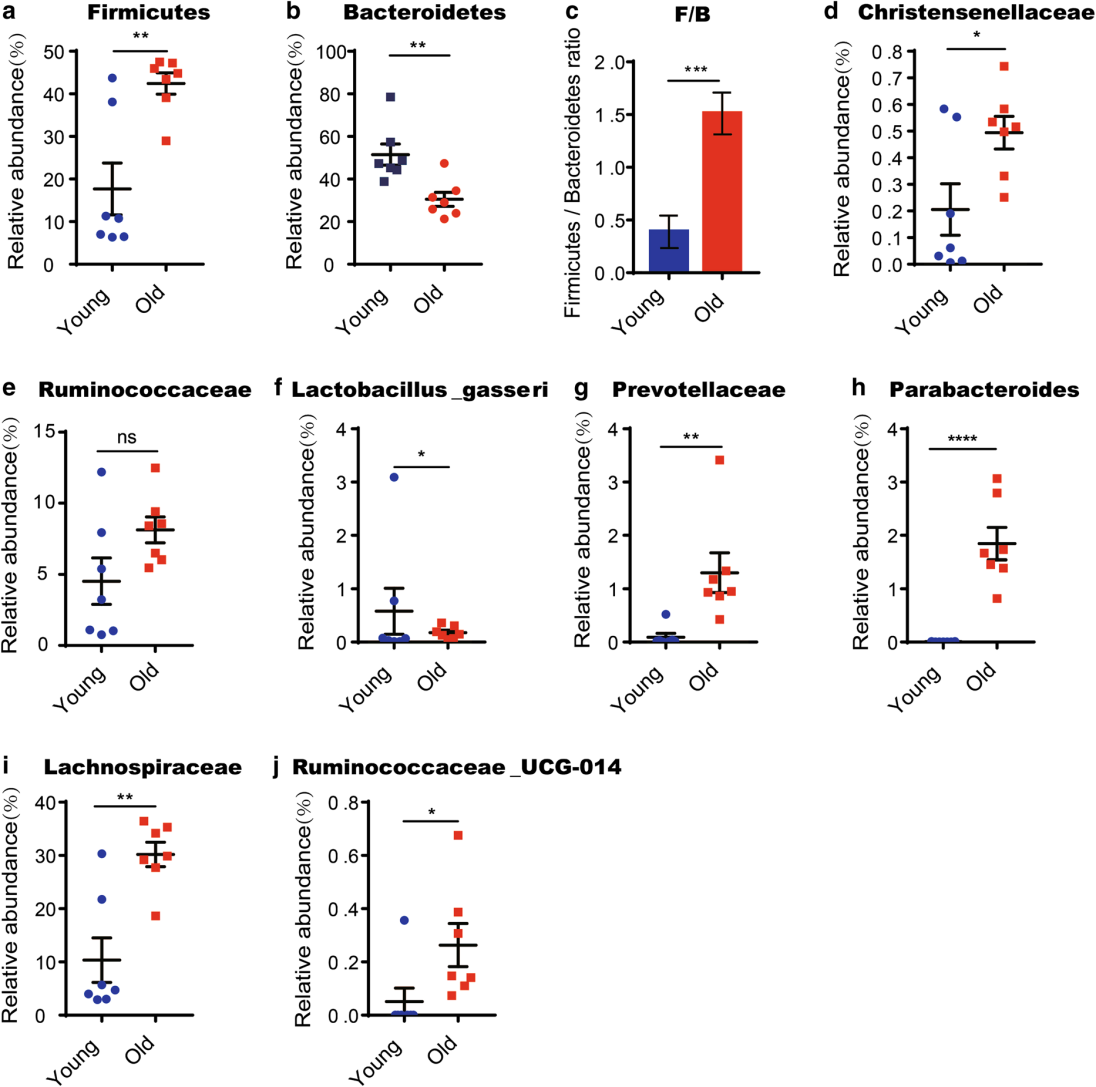
Lipid-promoting and pro-inflammatory bacteria are enriched in aging mice. Fecal samples of 2 months old (young) and 20–22 months old (old) mice were collected for analysis (n = 7 samples per group). **a**, **b**, **d**–**j** Relative abundance based on OTUs of intestinal bacteria taxa which are significantly changed in old mice. Note that these taxa were all lipid-promoting or pro-inflammatory bacteria. **c** Ratio of Firmicutes/Bacteroidetes based on relative abundance of OTUs. Note a significant increase in the old mice compared to the young ones. Results were displayed as mean ± SEM. *p < 0.05; **p < 0.01; ***p < 0.001; ****p < 0.0001 by unpaired two-tailed Student’s t test

**Fig. 3:**
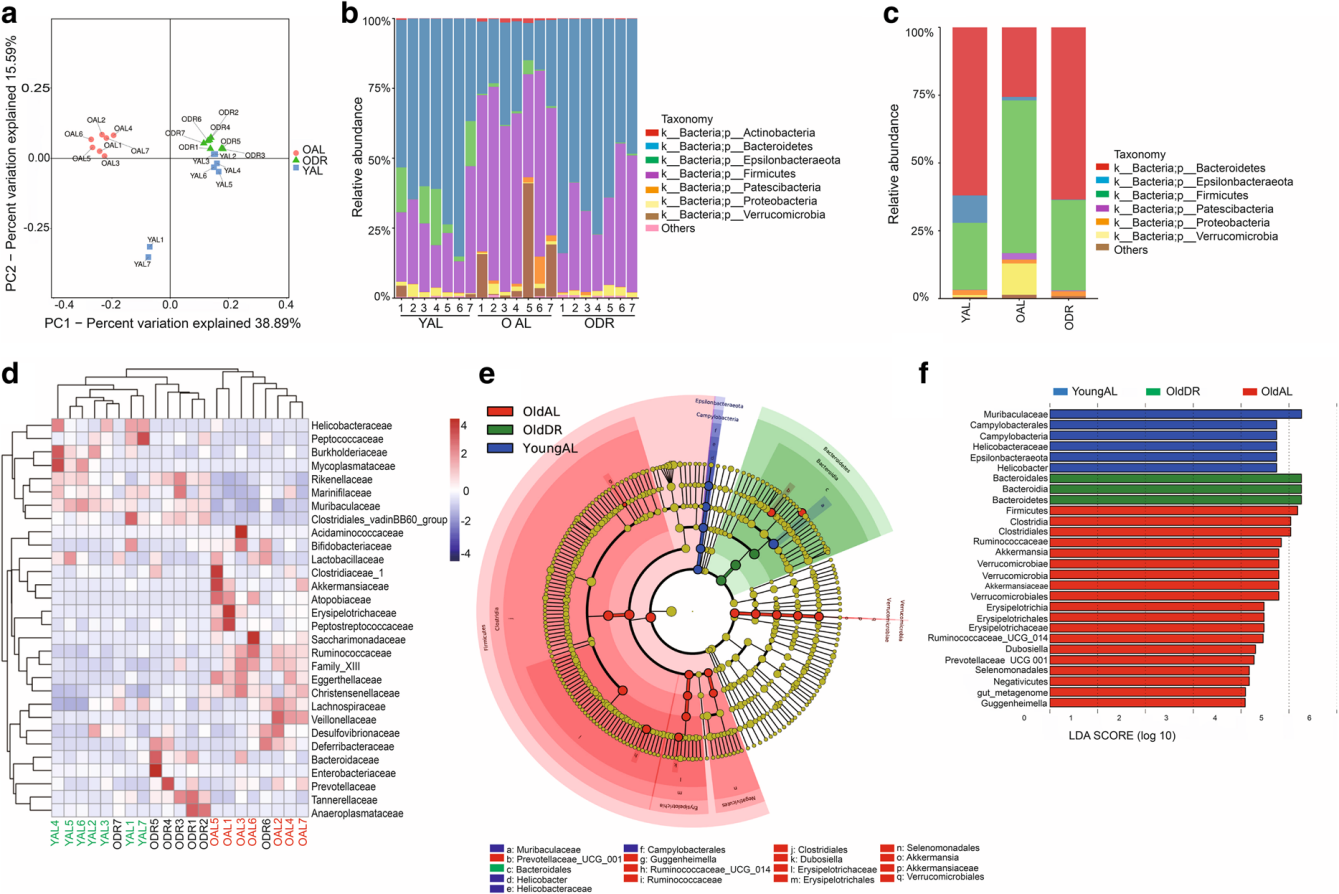
Short-term DR in old mice rejuvenates aging induced structural rearrangement of gut microbiota. 20–22 months old mice were treated with DR or AL diet for 2 months. Fecal samples of the following groups were collected for analysis: YAL (2 months old mice fed with ad libitum), OAL (22–24 months old mice fed with ad libitum), and ODR (22–24 months old mice pre-treated with DR for 2 months before sample collection) (n = 7 samples per group). **a** β-diversity analysis. The results of unweighted UniFrac PCoA of indicated groups were shown. **b** Relative abundance of bacteria at phylum level of individual sample based on OTUs. **c** The ratio of relative abundance at phylum level based on the average OTUs in each group. **d** Heatmap showing clustering of each sample at family level based on the relative abundance of OTUs. Note that hierarchical clustering shown that samples of ODR and YAL tend to cluster together. **e** Taxonomic cladogram from LEfSe showing differences in fecal taxa. Dot size is proportional to the abundance of the taxon. Letters correspond to the following taxa: a: Muribaculaceae, b: Prevotellaceae_UCG_001, c: Bacteroidales, d: Helicobacter, e: Helicobacteraceae, f: Campylobacterales, g: Guggenheimella, h: Ruminococcaceae_UCG_014, i: Ruminococcaceae, j: Clostridiales, k: Dubosiella, l: Erysipelotrichaceae, m: Erysipelotrichales, n: Selenomonadales, o: Akkermansia, p: Akkermansiaceae, q: Verrucomicrobiales. **f** LDA scores computed for differentially-abundant taxa in the fecal microbiomes of young (blue) old DR (green) and old AL (red). Length indicates effect size associated with a taxon. p = 0.05 for the Kruskal–Wallis test; LDA score > 2

**Fig. 4:**
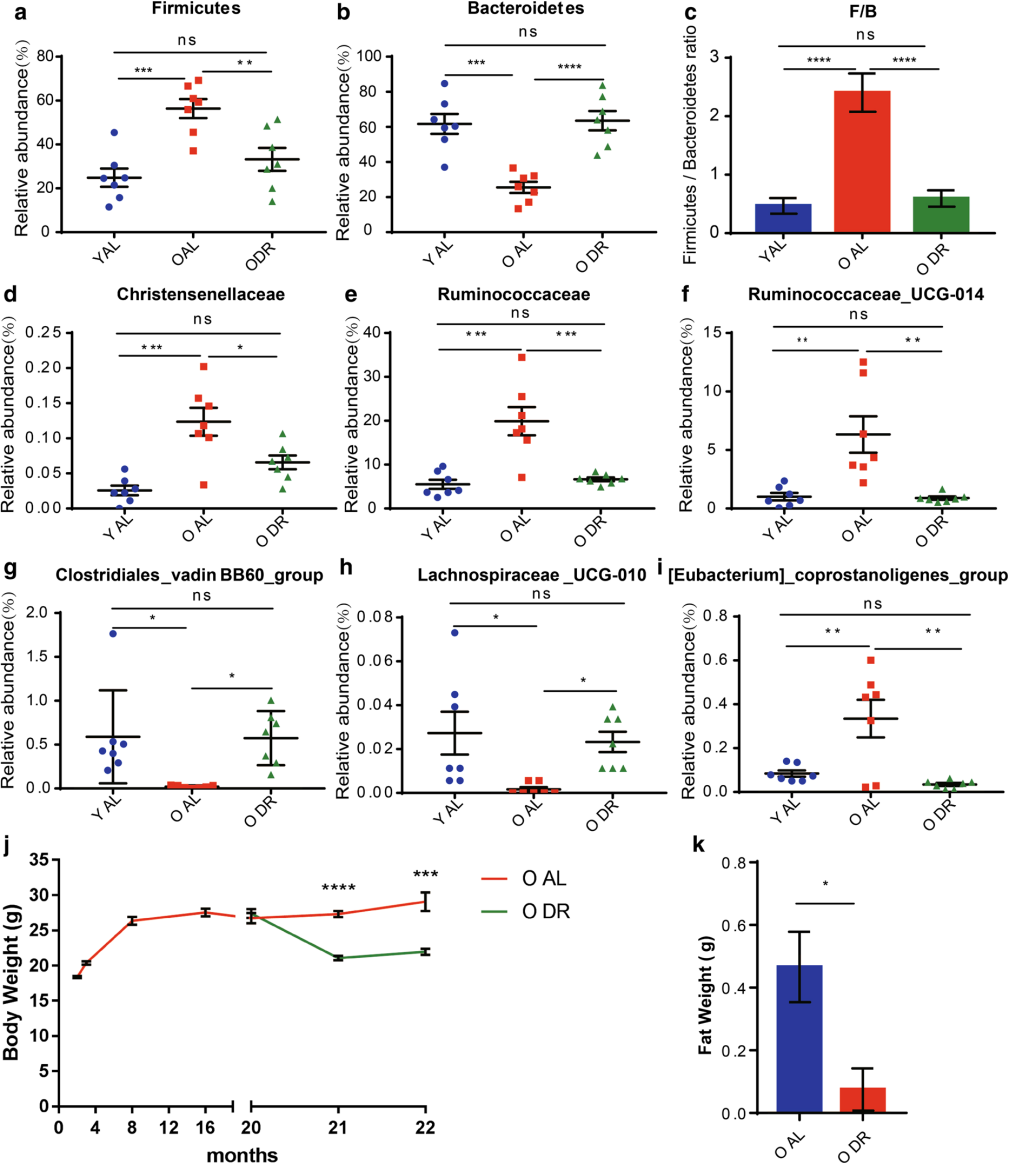
Short-term DR reverted compositional alterations of bacterial taxa associated with obesity and inflammation in aging mice. 20–22 months old mice were treated with DR or AL diet for 2 months. Fecal samples of the following groups were collected for analysis: YAL (2 months old mice fed with ad libitum), OAL (22–24 months old mice fed with ad libitum), and ODR (22–24 months old mice pre-treated with DR for 2 months before sample collection) (n = 7 samples per group). **a**, **b**, **d**–**i** Relative abundance based on OTUs of intestinal bacteria taxa. **c** Ratio of Firmicutes/Bacteroidetes based on relative abundance of OTUs. Note that DR significantly rejuvenated all alterations of indicated taxa in aging mice. **j**, **k** Body and belly fat weight of indicated groups. Note a significant reduction upon DR. Results were displayed as mean ± SEM. *p < 0.05; **p < 0.01; ***p < 0.001; ****p < 0.0001 by unpaired two-tailed Student’s t test

